# An Increase in Postural Load Facilitates an Anterior Shift of Processing Resources to Frontal Executive Function in a Postural-Suprapostural Task

**DOI:** 10.3389/fnhum.2016.00420

**Published:** 2016-08-19

**Authors:** Cheng-Ya Huang, Gwo-Ching Chang, Yi-Ying Tsai, Ing-Shiou Hwang

**Affiliations:** ^1^School and Graduate Institute of Physical Therapy, College of Medicine, National Taiwan UniversityTaipei City, Taiwan; ^2^Physical Therapy Center, National Taiwan University HospitalTaipei, Taiwan; ^3^Department of Information Engineering, I-Shou UniversityKaohsiung City, Taiwan; ^4^Institute of Allied Health Sciences, College of Medicine, National Cheng Kung UniversityTainan City, Taiwan; ^5^Department of Physical Therapy, College of Medicine, National Cheng Kung UniversityTainan City, Taiwan

**Keywords:** dual-task, graph analysis, functional connectivity, event-related potential, network-based statistics

## Abstract

Increase in postural-demand resources does not necessarily degrade a concurrent motor task, according to the adaptive resource-sharing hypothesis of postural-suprapostural dual-tasking. This study investigated how brain networks are organized to optimize a suprapostural motor task when the postural load increases and shifts postural control into a less automatic process. Fourteen volunteers executed a designated force-matching task from a level surface (a relative automatic process in posture) and from a stabilometer board while maintaining balance at a target angle (a relatively controlled process in posture). Task performance of the postural and suprapostural tasks, synchronization likelihood (SL) of scalp EEG, and graph-theoretical metrics were assessed. Behavioral results showed that the accuracy and reaction time of force-matching from a stabilometer board were not affected, despite a significant increase in postural sway. However, force-matching in the stabilometer condition showed greater local and global efficiencies of the brain networks than force-matching in the level-surface condition. Force-matching from a stabilometer board was also associated with greater frontal cluster coefficients, greater mean SL of the frontal and sensorimotor areas, and smaller mean SL of the parietal-occipital cortex than force-matching from a level surface. The contrast of supra-threshold links in the upper alpha and beta bands between the two stance conditions validated load-induced facilitation of inter-regional connections between the frontal and sensorimotor areas, but that contrast also indicated connection suppression between the right frontal-temporal and the parietal-occipital areas for the stabilometer stance condition. In conclusion, an increase in stance difficulty alters the neurocognitive processes in executing a postural-suprapostural task. Suprapostural performance is not degraded by increase in postural load, due to (1) increased effectiveness of information transfer, (2) an anterior shift of processing resources toward frontal executive function, and (3) cortical dissociation of control hubs in the parietal-occipital cortex for neural economy.

## Introduction

Postural control is a continuum raging from “controlled to automatic” processing, depending on the level of postural demand and the capacity of attentional resources (Stins et al., 2009; Boisgontier et al., 2013). Maintenance of posture with bilateral stance on a stable surface is an automatic process that requires minimal attentional resources to stabilize the center of gravity of the postural system within the limits of the sway range. When stance difficulty increases, the postural task shifts to a controlled process, manifested with an enhanced postural regularity (Donker et al., 2007; Sarabon et al., 2013). Parallel loading of two component tasks, posture and supraposture tasks, results in an intricate trade-off for central resource allocation (Temprado et al., 2001), depending on the task priority (Levy and Pashler, 2001), response compatibility (Stelzel et al., 2006), relative task difficulty of the two concurrent tasks (Huang and Hwang, 2013), and so on. For some postural-suprapostural dual-tasking, such as golf putting and surgery, withdrawing attention from postural control could help to maximize the precision of the added motor task (Balasubramaniam et al., 2000; Stoffregen et al., 2007). Postural sway is less regular in the dual-task condition than in a single postural task (Donker et al., 2007; Kuczyński et al., 2011). In this context, at least two critical issues with the limited central resource arise. The first issue is that relative cost of postural-suprapostural performance varies with stance difficulty, as differently predicted by the resource-competition model (Woollacott and Shumway-Cook, 2002) and the adaptive resource-sharing model (Mitra, 2004; Mitra and Fraizer, 2004). However, direct neural mechanism regarding to how the brain reorganizes functional networks is largely unknown. Second, most research designed to investigate the neural control of a postural-suprapostural task has employed a concurrent cognitive task as part of the dual-task configuration. Traditional dual-task setups and a postural-suprapostural task with a suprapostural motor goal are very likely to produce different types of resource competition. The reason is that the task quality of a suprapostural motor task must take kinematical advantages of stance stability (Wulf et al., 2004; Stoffregen et al., 2007; Huang and Hwang, 2013), whereas a postural-suprapostural task with a cognitive goal typically has low response compatibility between the two component tasks (Weeks et al., 2003).

The fronto-parietal brain network is a flexible hub of dual-task control (Cole et al., 2013), although the role of the frontal and parietal areas in a dual-task is still debatable. Some neuroimaging studies have reported greater activation of the frontal or prefrontal areas during dual-task trials than during single-task trials (D'Esposito et al., 1995; Collette et al., 2005), whereas others have revealed no specific frontal or prefrontal activation in the dual-task condition (Klingberg, 1998; Adcock et al., 2000; Bunge et al., 2000). In other studies, dual-tasking, as compared to both individual visual and auditory single tasks, activated a predominantly parietal network in the right hemisphere (Deprez et al., 2013), whereas simultaneous car driving and language comprehension suppressed parietal activation in reference to two single tasks (Just et al., 2008). In addition to a paradigm-specific interaction between component tasks (Salo et al., 2015), one of the most appealing explanations to reconcile those seemingly paradoxical results is that a dual-task may not necessarily recruit additional cortical areas; it may instead alter the interactions of the frontal/prefrontal areas with other cortical regions [such as parietal (Gontier et al., 2007) and premotor areas (Marois et al., 2006)]. Consequently, it is more important to examine changes in the inter-regional connectivity than to investigate changes in regional excitability of a dual-task by referencing the baseline activity of a single task.

Recently, graph theoretical analysis has been developed to characterize the topology of inter-regional connectivity and the efficacy of information transmission in brain networks, with important implications for adaptive or pathological changes in brain function (Reijneveld et al., 2007; Bullmore and Sporns, 2012). As postural-suprapostural behaviors involve information mastery potentially contingent upon the fronto-parietal network (Huang and Hwang, 2013), challenging postural sets could affect network connectivity for static stance, compromising the wiring-cost minimization, and postural load increment to achieve a suprapostural goal. Within the brain connectome context, this study aimed to extend the limited previous work by exploring the brain connectome in a particular postural-suprapostural task, when stance difficulty increases. This increase in stance difficulty must be associated with resource allocation of the brain, especially that of the fronto-parietal network, so that suprapostural motor performance and stance stability are jointly optimized. This exploratory study hypothesized that concurrent force-matching from a stabilometer stance would lead to changes in the inter-regional connectivity and the efficacy of information transfer, as compared to force-matching from a level-surface stance.

## Materials and methods

### Subjects

The study was conducted with 14 healthy right-handed volunteers (7 males, 7 females; mean age: 23.8 ± 3.8 years) from a university campus. All subjects were asked to abstain from stimulants (such as cigarettes, alcohol, and caffeine) for 24 h before the experiment. All subjects were volunteers naive to the purpose of the experiments and received no reimbursement. The experiment was conducted in accordance with the Declaration of Helsinki and with the approval of the local ethics committee (National Taiwan University Hospital Research Ethics Committee; no. 201312077RINC), and the subjects took part after signing personal consent forms.

### Procedures

**Figure 1:**
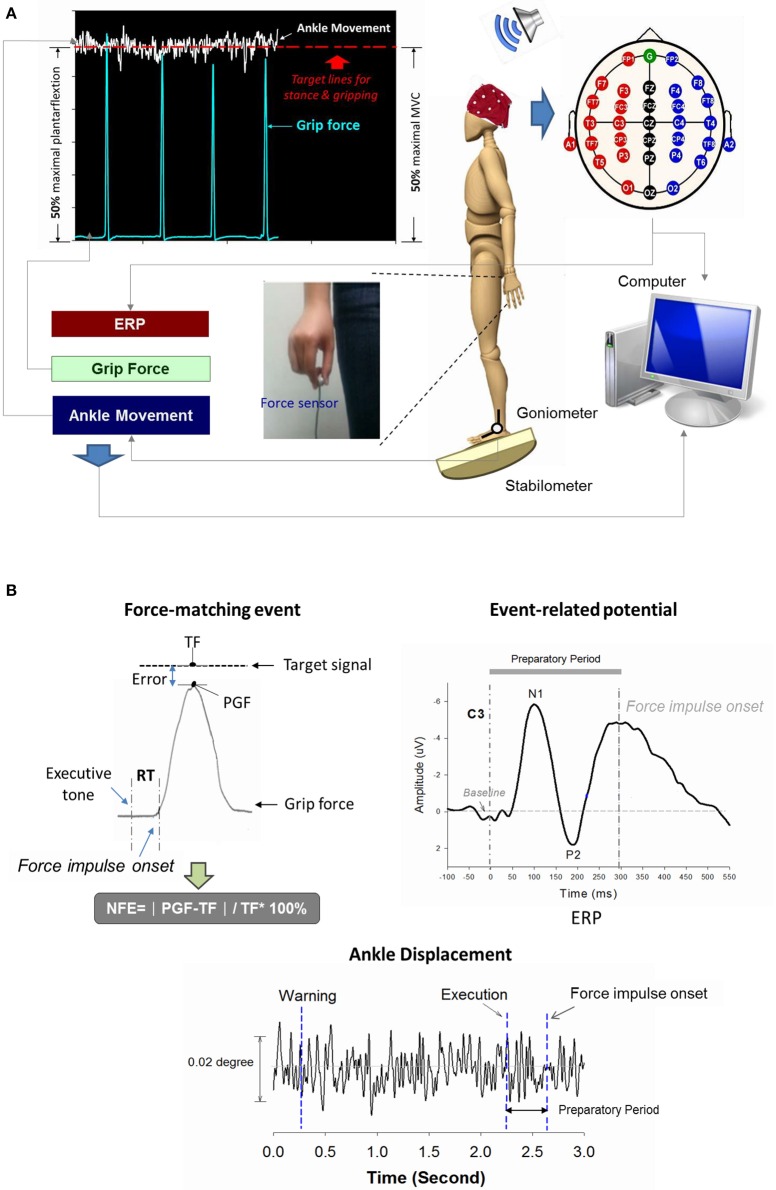
**Schematic illustration of experimental setup (A) and physiological data (B)**. Real-time display of precision grip force, ankle displacement, and target signals for concurrent force-matching and postural tasks. By separate scale-tuning of the manual force target and postural target, the target signals of both postural and force-matching tasks could be displayed in an identical position on the monitor. Suprapostural performance was assessed with the reaction time (RT) and normalized force error (NFE) of a force-matching act. The event-related potential (ERP) of the force-matching act was recorded with scalp electroencephalography. ERP between the executive tone and onset of the force-impulse profile was denoted as preparatory ERP, composed of N1 and P2 components. TF, target force; PGF, peak grip force.

Before the experiment, the maximum voluntary contraction (MVC) of the right thumb-index precision grip and the maximal anterior tilt angle during stabilometer stance of each participant were determined respectively. For each participant, there were two experimental conditions for concurrent postural and motor tasks with different postural challenges (level-surface stance vs. stabilometer stance). The participants were required to conduct a thumb-index precision grip to couple a target line of 50% MVC force in response to auditory cues (force-matching task) while standing on a level surface or a tilted stabilometer. The two conditions were varied in a random order. For the level-surface condition, the participants were instructed to execute the force-matching task as accurately as possible while standing on a level surface. Therefore, the participants focused the majority of their attention on the force-matching task and maintained the upright posture automatically. For the stabilometer condition, the participants performed the force-matching task while standing on a stabilometer [a wooden platform (50 × 58 cm) with a curved base (height: 25 cm)]. They were instructed to execute the force-matching task as accurately as possible while maintaining the stabilometer at 50% of the maximal anterior tilt with minimal ankle movement (Figure 1A). Therefore, the participants had to pay attention simultaneously to both the force-matching task and postural maintenance. In the stabilometer condition, the subjects were provided with on-line visual feedback regarding the ankle displacement and force output on a computer screen 60 cm in front of them at the participants' eye-level. The target signals for force-matching and posture were presented at the same vertical position of the monitor to reduce the visual load during the concurrent tasking. With visual feedback, the participants could minimize fluctuations of the ankle and force-error in reference to the target angle at all times. In the level-surface condition, only the force-matching related visual feedback was provided. We understood that relative task difficulty could affect the reciprocal effect and task outcome of a postural-suprapostural task. Therefore, the target force for the concurrent motor task and target angle for the postural task were empirically selected based on our previous experiment (Hwang and Huang, 2016; Hung et al., in press). A high target force for force-matching of over 50% MVC and a tilting angle of the stabilometer plate >50% of the maximal anterior tilt were not suitable for repeated measures of event-related potential because of the potential fatigue effect. Moreover, the present combination of tasks was intended to provide a unique dual-task situation that would prevent a marked reduction in force-matching performance due to stabilometer stance for the majority of young healthy adults in the laboratory.

The force-matching act was guided by warning and executive tones, with a total of 14 warning-executive signal pairs in an experimental trial. A warning tone (an 800 Hz tone lasting for 100 ms) was randomly presented at different intervals of 1.5, 1.75, 2, 2.25, 2.5, 2.75, or 3 s before an executive tone (a 500 Hz tone lasting for 100 ms). The interval between the end of the executive tone and the beginning of the next warning tone was 3.5 s. Upon hearing the executive tone, the participants started a quick thumb-index precision grip (force impulse duration < 0.5 s) to quickly couple the peak precision-grip force with the force target on the monitor. There were six trials of the postural-suprapostural dual-task for each stance condition, and each trial was composed of 14 precision grips.

### Experimental setting

An electrogoniometer (Model SG110, Biometrics Ltd, UK) was used to record the angular motion of the right (dominant) ankle joint. The electrogoniometer consisted of a 12-bit analog-to-digital converter box and 2 sensors for measuring their relative positions in space. One sensor was placed on the dorsum of the right foot between the second and third metatarsal heads, and the other sensor was fastened along the midline of the middle third of the anterior aspect of the lower leg. The level of force-matching was recorded with a load cell (15-mm diameter × 10-mm thickness, net weight = 7 grams; Model: LCS, Nippon Tokushu Sokki Co., Japan) mounted on the right thumb. The load cell was connected to a distribution box by a thin wire that could not provide stable mechanical support for the postural stance via the grip force apparatus. The auditory stimuli and target signals for conducting the force-matching and postural subtasks were generated with LabVIEW software (National Instruments, Austin, TX, USA). Thirty-two Ag-AgCl scalp electrodes (Fp_1/2_, F_z_, F_3/4_, F_7/8_, FT_7/8_, FC_z_, FC_3/4_, C_z_, C_3/4_, CP_z_, CP_3/4_, P_z_, P_3/4_, T_3/4_, T_5/6_, TP_7/8_, O_z_, O_1/2,_ and A_1/2_) with a NuAmps amplifier (NeuroScan Inc., EI Paso, TX, USA) were used to register scalp voltage fluctuations in accordance with the extended 10–20 system. The ground electrode was placed along the midline ahead of F_z_. Electrodes placed above the arch of the left eyebrow and below the eye were used to monitor eye movements and blinks. The impedances of all the electrodes were below 5 kΩ and were referenced to linked mastoids of both sides. All physiological data were synchronized and digitized at a sample rate of 1 kHz.

### Data analyses

#### Behavior data

Reaction time (RT) and force error of force-matching was used to represent suprapostural performance in the present study. The RT of force-matching was denoted as the timing interval between the executive tone and the onset of grip force. The onset of grip force was defined as the force impulse profile exceeding the mean plus 3 times the standard deviation of the baseline activity of the force profile (500 ms before and after each warning tone). The RT of each force-matching trial was averaged across trials for each participant in the level-surface and stabilometer conditions. Force error of each force impulse was determined by normalized force-matching error (NFE), denoted as $\frac{\left|\text{PGF }-\text{ TF}\right|}{\text{TF}}\times 100\text{\%}$ (where PGF: peak grip force; TF: target force; Figure 1B). The NFEs of all force-matching events were also averaged across trials for each participant in the level-surface and stabilometer conditions. On the other hand, the kinematic properties of ankle movement fluctuations during the interval between the executive tone and the onset of the force impulse profile were used to represent postural performance. The amplitude and regularity of the ankle movement fluctuations were assessed with root mean square and sample entropy (SampEn) after down-sampling of the kinematic data to 125 Hz. SampEn is a popular entropy measure used to characterize the temporal aspects of the variability of biological data, with high consistency and less sensitivity to short data length (Richman and Moorman, 2000; Yentes et al., 2013). A SampEn close to 0 represents greater regularity, while a value near 2 represents higher irregularity. A higher postural irregularity indicates less attentional resources allocated to postural control and thus more autonomous processing (Donker et al., 2007; Kuczyński et al., 2011). The mathematical formula for *SampEn* was

$$\begin{array}{l} SampEn\left(m,r,N\right)=\ln\;\left(\frac{\overset{N\;-\;m}{\underset{i\;=\;1}{\sum }}n_{i}^{m}}{\overset{N\;-\;m\;-\;1}{\underset{i\;=\;1}{\sum }}n_{i}^{m\;+\;1}}\right)=\ln\;\left(\frac{n_{n}}{n_{d}}\right) \end{array}$$

where *N* is the total data point number. In this study, *m* equaled 3 and the tolerance range of *r* was 0.15 × the standard deviation of the standardized ankle movement fluctuations. For the level-surface condition, the data of the absolute ankle joint angle were used for amplitude and *SampEn* measurement; for the stabilometer condition, the data of the mismatch between the absolute ankle joint angle and the target line were used for amplitude and *SampEn* measurement.

#### Functional connectivity assessment

The DC shift and artifacts of electrical noise of each channel were conditioned with third-order trend correction and a low pass filter (40 Hz/48 dB roll-off) over the entire set of recorded data in off-line analysis. The conditioned EEG data were then segmented into epochs of 700 ms, including 100 ms before the onset of each execution signal. Each epoch was corrected with the NeuroScan 4.3 software program (NeuroScan Inc., EI Paso, TX, USA) to remove artifacts (such as excessive drift, eye movements, or blinks) in reference to baseline activities at the pre-stimulus interval. Poor epochs were also discarded by visual inspection (rejection rate of inappropriate trials: <10%). The remaining artifact-free epochs were averaged for an experimental trial in the level-surface and stabilometer conditions.

**Figure 2:**
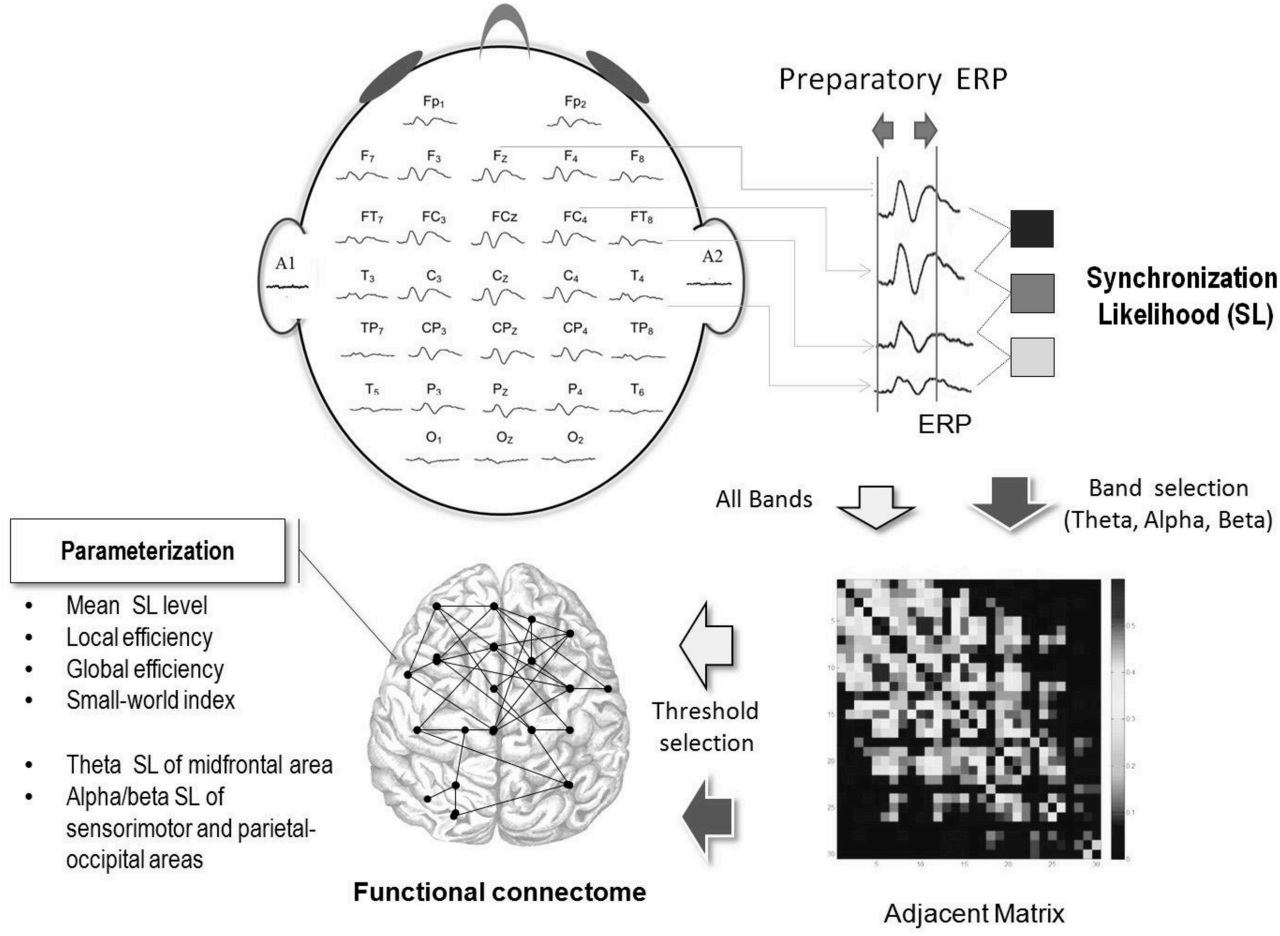
**Workflow of cortical network construction with ERP in the preparatory period**. Functional connectivity was characterized with the synchronization likelihood (SL) of the paired preparatory ERP of different recording electrodes. The adjacent matrix represents the strengths of inter-electrode synchronization likelihood following appropriate threshold selection. With the adjacent matrix, connectomes in the preparatory period in the level-surface, and stabilometer conditions were established.

Since brain networks can be coupled in a highly non-linear manner (Pijnenburg et al., 2008), the synchronization likelihood (SL) was used to assess the degrees of linear and non-linear dimensions of EEG coupling within cortical networks (Leistedt et al., 2009; Boersma et al., 2011). Theoretically, SL takes into account the recurrences of state space vectors occurring at the same moment that are converted from two time series of interest (Boersma et al., 2011). An SL close to 0 indicates no coupling, whereas an SL of 1 indicates complete coupling. For brevity, detailed descriptions of SL calculation (Stam and van Dijk, 2002; Stam et al., 2003) and parameter settings (Montez et al., 2006) can be found in previous works. Because we were interested in cortical modulation during preparation process for a postural-suprapostural task, we selected the duration of averaged epochs between the executive tone and force-matching onset for SL analysis. A square 30 × 30 SL adjacent matrix was obtained by computing the SL of ERP data from all pairwise combinations of channels in the preparatory period (Figures 1B, 2). In the Figure 1B, the 0 of ERP plot represents the onset of the executive signal. Each entry in the SL adjacent matrix represented the connectivity strength within the functional networks. For each participant, the overall SL adjacent matrix from 6 experimental trials in the level-surface or stabilometer condition was averaged. As the choice of the threshold is fairly arbitrary in the literature, we built functional connectomes across various SL thresholds from 0.1 to 0.9. The SL adjacent matrix was rescaled with the proportion of strongest weights, such that all other weights below a given threshold (including SL on the main diagonal) were set to 0. For instance, when the threshold value was 0.1, only the top 10% of the strongest weights in the SL adjacent matrix were considered to determine the network properties of the functional connectome. The mean SL of all the 32 recording sites, frontal (F_z_, F_3_, F_4_, F_7_, and F_8_), sensorimotor (C_3_, C_z_, C_4_, CP_3_, CP_z_, and CP_4_), and parietal-occipital areas (P_3_, P_z_, P_4_, O_1_, O_z_, and O_2_), were determined for the level-surface and stabilometer conditions.

Using a causal finite impulse response (FIR) filter (24 dB/octave roll-off), we digitally filtered the ERP signal into the classic frequency bands in the theta (4–8 Hz), upper alpha (10–13 Hz), and beta (13–35 Hz) ranges. Spectral connectivity analysis was performed following the construction of theta and beta SL adjacent matrices with the conditioned ERP signal of both specific bands in the preparatory period. As action monitoring and planning for visuomotor tasks are reported to be linked to the mid-frontal theta rhythm (4–8 Hz; Luu et al., 2004; Armbrecht et al., 2012), the SL in the theta band that connected the mid-frontal areas (F_z_ and FC_z_) and other scalp electrodes was used to examine stance-related differences in central executive function (Tanaka et al., 2009). Another research interest was the SL in the upper alpha (10–13 Hz) and beta bands (13–35 Hz) that connected sensorimotor (C_3_, C_z_, C_4_, CP_3_, CP_z_, and CP_4_) or parietal-occipital areas (P_3_, P_z_, P_4_, O_1_, O_z_, and O_2_), as oscillatory changes in these areas are related to preparation for fine motor/postural control (MacKay and Mendonca, 1995; Brovelli et al., 2004; Babiloni et al., 2008) and early perception information processing (Nierhaus et al., 2015). Especially, upper alpha power is more associated with movement performance than low alpha power is (Babiloni et al., 2008). The calculation of SL was accomplished with functions of HERMES for Matlab (Niso et al., 2013).

#### Graph theoretical analysis

Graph theoretical analysis was conducted with weighted network measures to best utilize the weight information. In terms of SL adjacent matrixes at various threshold values, the mean clustering coefficient (*C*_*w*_), local coefficient (*E*_*loc*_), global coefficient (*E*_*glob*_), and small-world index (*sigma*, σ) of the resulting graphs were determined. Functionally, *C*_*w*_, *E*_*loc*_, and *E*_*glob*_ are metrics of information flow in a brain network. The clustering coefficient ($C_{w}^{i}$) for a vertex *i* quantifies the proportion of its neighboring vertices *j* that are connected to each other. The clustering coefficient $C_{w}^{i}$ of vertex *i* was denoted as $C_{w}^{i}=\;\frac{\underset{j\;\neq \;i}{\sum }\underset{k\;\neq \;i,j}{\sum }w_{ij}w_{ik}w_{jk}}{\underset{j\;\neq \;i}{\sum }\underset{k\;\neq \;i,j}{\sum }w_{ij}w_{ik}}$. Topological mapping of the clustering coefficient was constructed with the clustering coefficients of all nodes. Another network metric, *E*_*loc*_ can be defined as $E_{loc}=\frac{1}{N}\underset{i\in G}{\sum }E\left(G_{i}\right)$, where *G*_*i*_ is the subgraph of the neighbors of a node *i* and *E(G*_*i*_*)* indicates the efficiency of the subgraph *G*_*i*_. *E*_*glob*_ was calculated with $E_{glob}=\frac{1}{N\left(N\;-\;1\right)}\underset{i\;\neq \;j\in G}{\sum }\frac{1}{L_{i,j}}$, where *L*_*i, j*_ is the shortest path length from node *i* to node *j*. Presuming that cortical regions under different electrodes exchange packets of information concurrently, *E*_*glob*_ is a quantitative measure of the efficiency of a parallel information transfer, with greater *E*_*glob*_ indicating better functional integration of brain networks. In contrast to *E*_*glob*_, which indexes a network property of functional integration (Rubinov and Sporns, 2010; Yu et al., 2013), *C*_*w*_ and *E*_*loc*_ reflect a network property of functional segregation (Rubinov and Sporns, 2010; Yu et al., 2013). By contrasting the random and regular networks of the same numbers of nodes and edges (Watts and Strogatz, 1998), the small-world index (σ) can provide the network's small-worldness regarding the balance of information flow between local segregation and global integration in a network (Watts and Strogatz, 1998). When σ is >1, the network exhibits small-world properties (Humphries et al., 2006; Stam et al., 2007a). The small-world index (σ) is mathematically formulated as σ = γ/λ. Here, γ = *C*_*w*_/*C*_*w_rand*_ and λ = *L*_*p*_*/L*_*p_rand*_. The *C*_*w*_ and *L*_*p*_ are the clustering coefficient and the characteristic path length of the functional network. The characteristic path length is formulated as $L_{p}=\frac{1}{n}\underset{i\in N}{\sum }L_{i}=\frac{1}{n}\underset{i\in N}{\sum }\frac{\sum j\in N,j\;\neq \;i,\wedge d_{i,j}}{n\;-\;1}$, where *L*_*i*_ is the average distance between mode *i* and all other nodes. Both *C*_*w_rand*_ and *L*_*p_rand*_ were obtained by averaging 50 populations of random networks. The parameterization of network properties was accomplished with functions of the Brain Connectivity Toolbox (Rubinov and Sporns, 2010).

#### Statistical analysis

For behavior data, paired *t*-test was used to examine the significance of differences between the level-surface and stabilometer conditions in normalized force-matching error (NFE), reaction time (RT), amplitude of ankle movement fluctuations (AMF_RMS), and sample entropy of ankle movement fluctuations (AMF_SampEn). Since these resemble behavior variables, paired *t*-test was used to contrast all the network parameters [mean SL of all electrode pairs (SL_all), in the frontal (SL_F), sensorimotor (SL_SM), parietal-occipital (SL_PO) areas, *E*_*glob*_, *E*_*loc*_, and small-world index (σ)] of the level-surface and stabilometer conditions across different threshold values. The significance of the stance effect on modulation of clustering coefficients and the mean SL of all electrode pairs was displayed with *t*-values on the basis of a paired difference test. The level of significance of the above-mentioned statistical analyses was set at *p* = 0.05. Network-based statistics were performed to identify spectral connectivity in the theta, upper alpha, and beta bands of the node pairs that significantly changed with variations in stance configuration. For this purpose, paired *t*-tests were independently performed at each synchronization value of the spectral bands of interest, and t-statistics larger than an uncorrected threshold of *t*_(13)_ = 3.012 (*p* = 0.005) were extracted into a set of supra-threshold connections. Then we identified all connected components in the adjacency matrix of the supra-threshold links and saved the number of links. A permutation test was performed 5000 times to estimate the null distribution of maximal component size, and the corrected *p*-value was calculated as the proportion of permutations for which the most connected components consisted of two or more links. Methodological details of network-based statistics are documented in Zalesky et al. (2010). Statistical analyses were performed in Matlab (Mathworks Inc. Natick, MA, USA) and SPSS v.19.0 (SPSS Inc. Chicago, IL, USA). All data are represented as mean ± standard error.

## Results

### Force-matching and stance performance

For the suprapostural task, the paired *t*-test revealed that the force-matching error (NFE: level-surface = 9.89 ± 0.78%; stabilometer = 10.01 ± 0.79%) and reaction time (RT: level-surface = 304.8 ± 9.6 ms; stabilometer = 310.7 ± 9.8 ms) of the force-matching task did not change with stance configuration (*p* > 0.05; Table 1). For the postural task, the magnitude of ankle movement fluctuations (AMF_RMS) was stance-dependent, for AMF_RMS of the stabilometer condition (0.151 ± 0.023) was greater than that of the level-surface condition (0.012 ± 0.001; *p* < 0.001). The sample entropy of ankle movement fluctuations (AMF_SampEn) was subject to postural load, for AMF_SampEn (0.382 ± 0.006) was lower in the stabilometer condition than in the level-surface condition (0.514 ± 0.013; *p* < 0.001).

**Table 1:** **Means and standard errors of force-matching and postural variables for the concurrent force-matching and postural tasks in the level-surface and stabilometer conditions**.

**Mean ± SE**	**Level-surface**	**Stabilometer**	**Statistics**
NFE (%)	9.89 ± 0.78	10.01 ± 0.79	*t*_(13)_ = −0.328, *p* = 0.748
RT (ms)	304.8 ± 9.6	310.7 ± 9.8	*t*_(13)_ = −1.720, *p* = 0.109
AMF_RMS (degree)	0.012 ± 0.001	0.151 ± 0.023^†††^	*t*_(13)_ = −6.138, *p* < 0.001
AMF_SampEn	0.514 ± 0.013	0.382 ± 0.006^†††^	*t*_(13)_ = 8.049, *p* < 0.001

NFE, normalized force-matching error; RT, reaction time for force-matching; AMF_RMS, root mean square value of ankle movement fluctuations; AMF_SampEn, sample entropy of ankle movement fluctuations;

††† *, stabilometer > level-surface, p < 0.001*.

### Global network metrics and inter-regional connectivity

**Figure 3:**
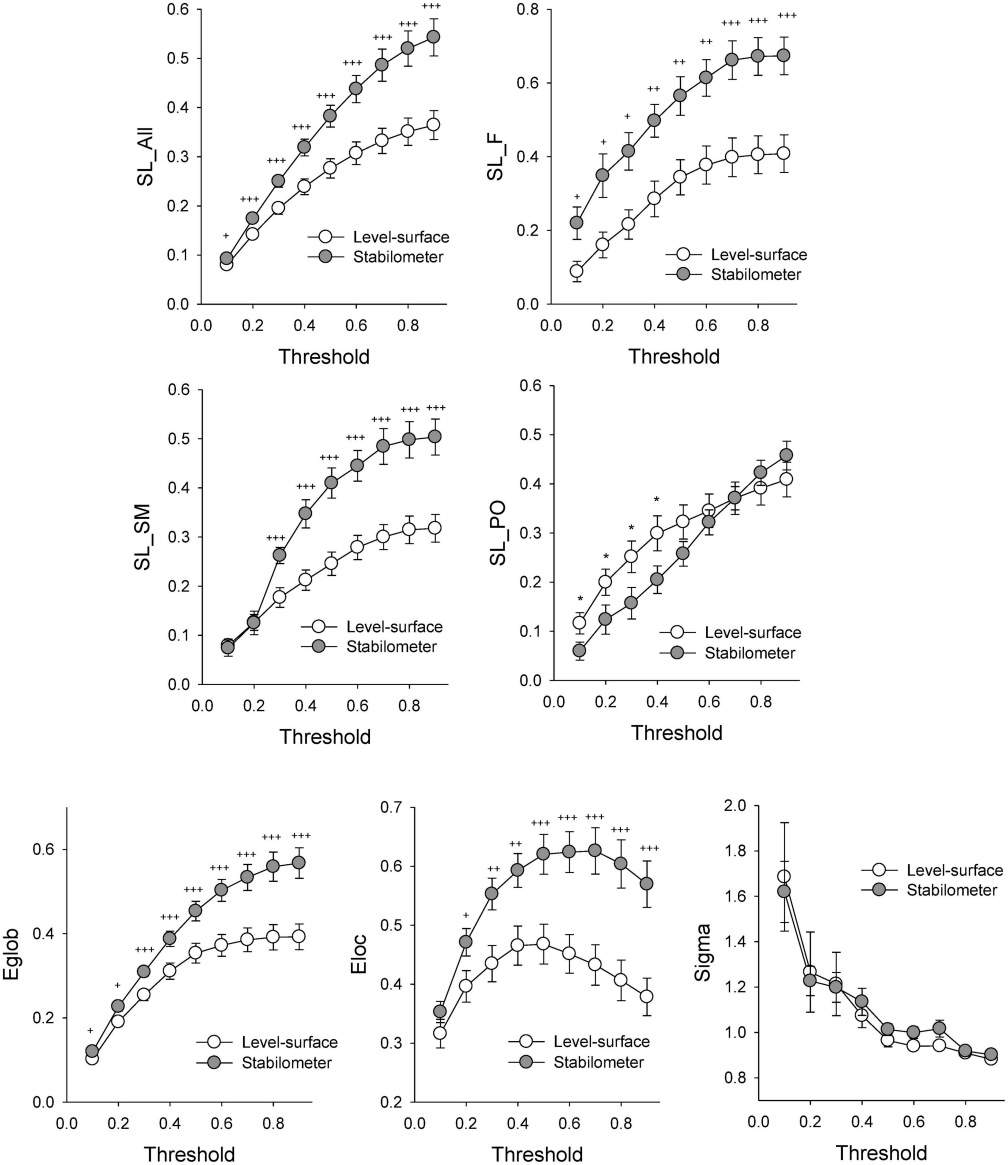
**The contrast of network parameters between the concurrent force-matching and postural tasks in the level-surface and stabilometer conditions at different threshold values**. *E*_*glob*_, global efficiency; *E*_*loc*_, local efficiency; *Sigma*, small-world index; ^*^, level-surface > stabilometer, *p* < 0.05; ^†^, stabilometer > level-surface, *p* < 0.05; ^††^, stabilometer > level-surface, *p* < 0.01; ^†††^, stabilometer > level-surface, *p* < 0.001.

**Figure 4:**
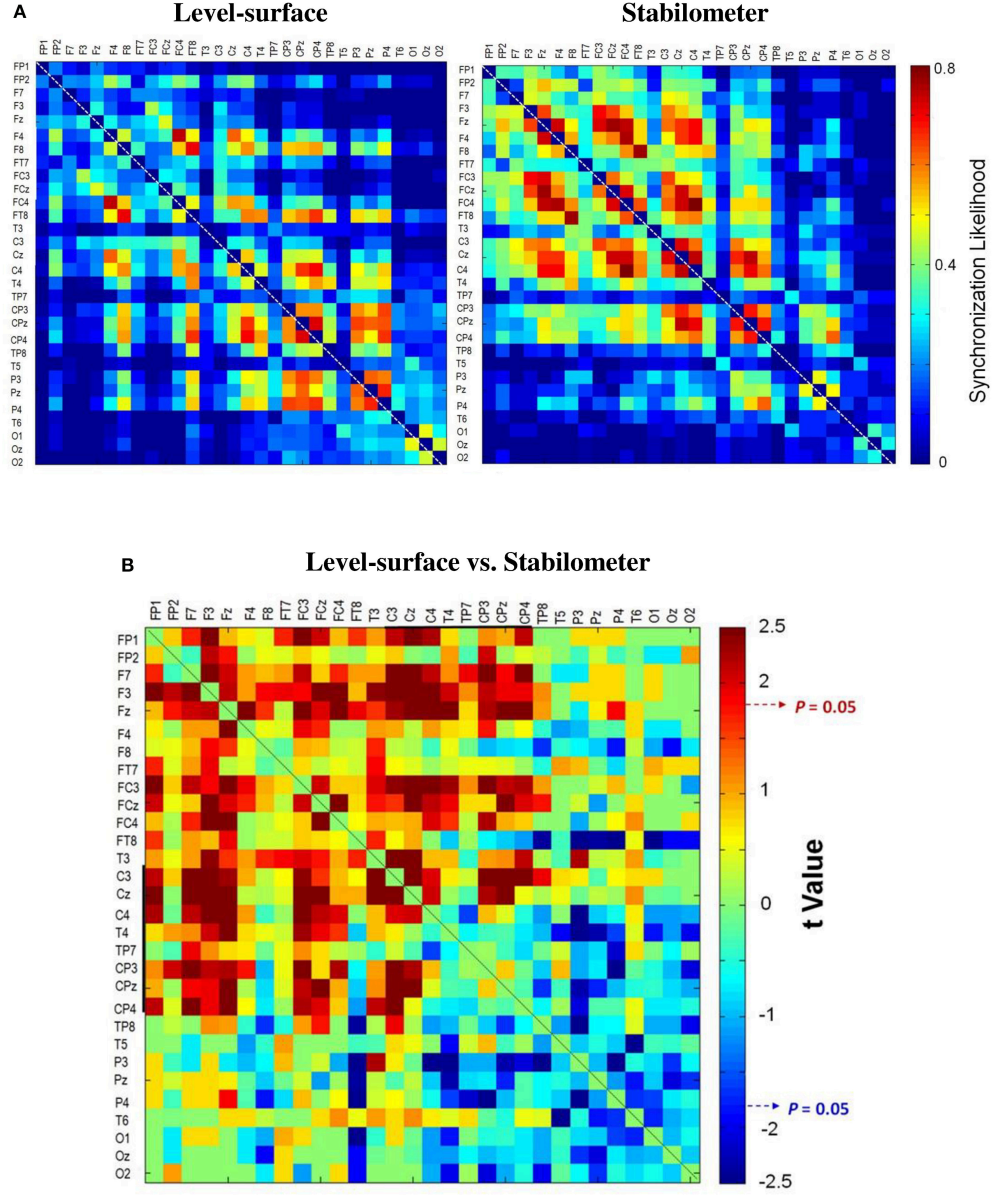
**The pooled adjacent matrix of synchronization likelihood (SL) of preparatory ERP for the concurrent force-matching and postural tasks in the level-surface and stabilometer conditions (threshold value = 0.3)**. **(A)** Population means of SL adjacent matrix in the surface (left) and stabilometer (right) conditions. **(B)** The adjacent matrix of *t*-values for contrasting SL between the level-surface and stabilometer conditions (*t* > 1.771, stabilometer SL > level-surface SL, *p* < 0.05; *t* < −1.771, level-surface SL > stabilometer SL, *p* < 0.05).

**Figure 5:**
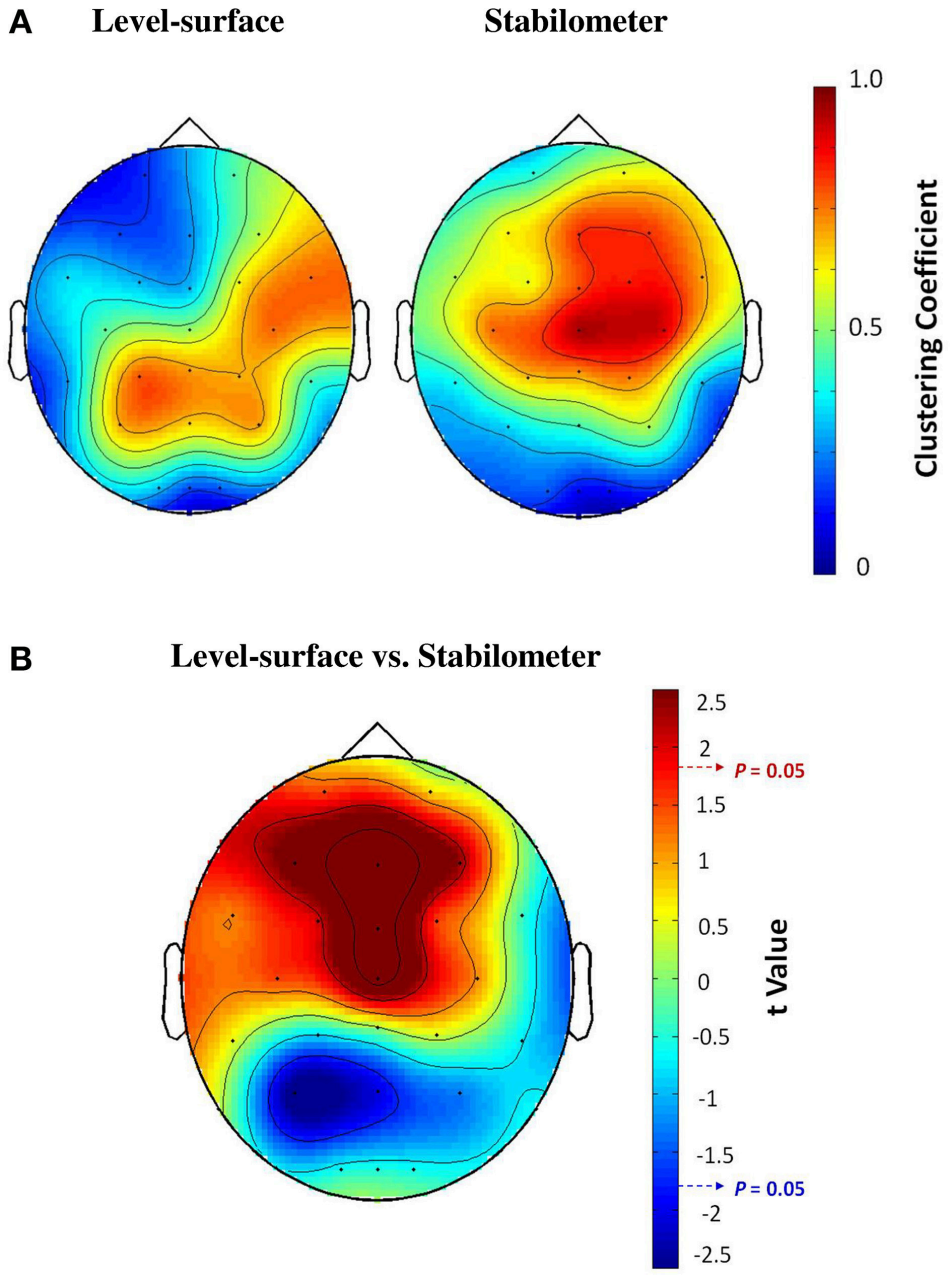
**(A)** The pooled topological mapping of clustering coefficients (*C*_*w*_) between concurrent force-matching and postural tasks in the level-surface and stabilometer conditions (threshold value = 0.3). **(B)** The stance-dependent difference in the distribution of clustering coefficients, in terms of the topography of *t*-values plotted on the scalp (*t* > 1.771, stabilometer *C*_*w*_ > level-surface *C*_*w*_, *p* < 0.05; *t* < −1.771, level-surface *C*_*w*_ > stabilometer *C*_*w*_, *p* < 0.05).

Figure 3 contrasts different network metrics as a function of threshold value between the level-surface and stabilometer conditions. For the majority of the threshold values, the global coefficient (*E*_*glob*_) and the local coefficient (*E*_*loc*_) in the stabilometer condition were significantly larger than those in the level-surface condition (*p* < 0.05), especially for the higher threshold values. However, the small-world index (σ) was stance-invariant for all threshold values (*p* > 0.05). In terms of synchronization likelihood, we found significant stance effects on inter-regional coupling of ERP in the preparatory stage. The mean values of the SL of all the electrode pairs in the frontal and sensorimotor areas (SL_F and SL_SM) were larger in the stabilometer condition than in the level-surface condition (*p* < 0.05). Mean SL in the parietal-occipital area (SL_PO) showed the reverse trend, with lower SL_PO in the stabilometer condition at lower threshold values (threshold = 0.1, 0.2, 0.3, and 0.4; *p* < 0.05). Overall, the mean SL of all the electrode pairs (SL_all) was enhanced in the stabilometer condition (*p* < 0.05). Except for the small-world index, the network parameters were stance-dependent when the threshold was set to 0.3 or 0.4. Figure 4 displays the population means of adjacent SL matrices in the two different stance conditions (threshold value = 0.3), as well as *t*-values for the examination of the stance effects on the adjacent matrices. In line with the patterned change in the mean level of SL, adjacent SL matrices in the frontal and sensorimotor areas were enhanced, whereas adjacent SL matrices in the parietal-occipital area were suppressed in the stabilometer condition. Figure 5A presents the pooled topology of the clustering coefficients (*C*_*w*_) for the level-surface and stabilometer conditions (threshold value = 0.3), which suggested different functional segregations between the two stance conditions from the standpoint of information flow. The concurrent postural and force-matching tasks in the level-surface condition exhibited a high probability of node connection to neighbors in the parietal lobes and the right fronto-temporal area, in contrast to high frontal *C*_*w*_ in the stabilometer condition. Figure 5B is a topological plot of *t*-values for contrasting the spatial distribution of *C*_*w*_ between the level-surface and stabilometer conditions. The stabilometer condition produced a higher *C*_*w*_ in the mid-frontal area but a lower *C*_*w*_ in the parietal area as compared to those in the level-surface condition (*p* < 0.05).

### Network-based statistics of spectral connectivity

**Figure 6:**
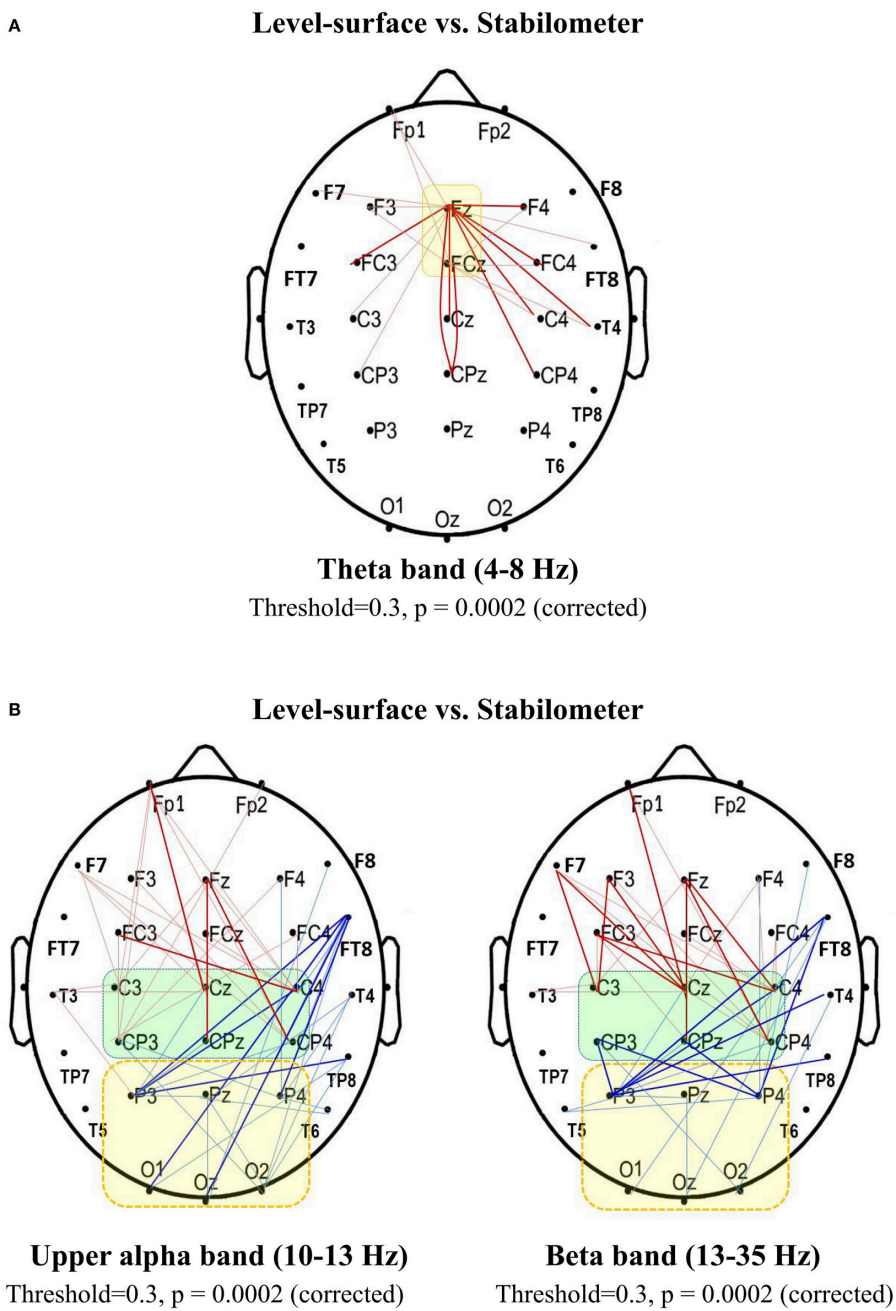
**Spectral connectivity analysis for contrasting wiring diagrams between the concurrent force-matching and postural tasks in the level-surface and stabilometer conditions (threshold value = 0.3). (A)** Synchronization likelihood in the theta band (4–8 Hz) that connects to the mid-frontal area (F_z_ and FC_z_). **(B)** Synchronization likelihood in the upper alpha (10–13 Hz) and beta band (13–35 Hz) of recording electrodes that connect to the sensorimotor (C_3_, C_z_, C_4_, CP_3_, CP_z_, and CP_4_) and parietal-occipital (P_3_, P_z_, P_4_, P_3_, O_1_, O_z_, and O_2_) areas (thin red line, stabilometer connectivity > level-surface connectivity, *p* < 0.05; bold red line, stabilometer connectivity of supra-threshold > level-surface connectivity of supra-threshold, *p* < 0.005; thin blue line, level-surface connectivity > stabilometer connectivity, *p* < 0.05; bold blue line, level-surface connectivity of supra-threshold > stabilometer connectivity of supra-threshold, *p* < 0.005).

Based on the supra-threshold connectivity and permutation test, network-based statistics revealed localized networks (i.e., connected and clustered components) with significantly stance-dependent SL-values in the theta, upper alpha, and beta bands (*p* = 0.0002, corrected). The contrast of stance-related average values of the spectral connectivity in the pairwise connections of interest is displayed in Figure 6. Theta connectivity (4–8 Hz) to the mid-frontal area (F_z_, FC_z_) was stronger in the stabilometer condition than in the level-surface condition (Figure 6A). On the other hand, there was a neat dichotomy of stance-related differences in upper alpha (10–13 Hz) and beta (13–35 Hz) connectivity to the sensorimotor (C_3_, C_z_, C_4_, CP_3_, CP_z_, and CP_4_) and parietal-occipital cortex (P_3_, P_z_, P_4_, O_1_, O_z_, and O_2_; Figure 6B). In comparison with the level-surface stance, the upper alpha and beta connectivity to the parietal-occipital cortex, especially the supra-threshold linkages from the right frontal-temporal (FT_8_), temporal (TP_8_, T_4_), and sensorimotor areas (CP_3_, CP_z_, and CP_4_; *p* < 0.005), was significantly suppressed with stabilometer stance (*p* < 0.05). However, the long-distance connectivity of the upper alpha and beta bands to the sensorimotor area (C_3_, C_z_, C_4_, CP_3_, CP_z_, and CP_4_), especially the supra-threshold linkages from the prefrontal and frontal areas (*p* < 0.005), was enhanced in the stabilometer condition.

## Discussion

As expected, the brain adopted a more controlled process for stabilometer stance in respond to increases in the magnitude and regularity of ankle movement. However, the accuracy and reaction time of suprapostural force-matching from stabilometer stance were not significantly affected by increases in postural threats. Our data did not support the facilitation of suprapostural performance by attention withdrawal from the postural task under the framework of resource competition (Cavanaugh et al., 2007; Donker et al., 2007; Derlich et al., 2011; Kuczyński et al., 2011). In fact, the combination of postural and suprapostural tasks of different task loads could result in a variety of performance outcomes, which are not always explainable with behavior contexts. The adaptive resource-sharing hypothesis (Mitra, 2004; Mitra and Fraizer, 2004) seems to be more appropriate for explaining the present observations, for concurrent force-matching was not affected by increasing attentional focus on the postural task. This preliminary study first revealed that brain reorganization under this particular circumstance involved (1) increased efficacy of information transmission, (2) anterior shift of processing resources, and (3) superior network economy in the preparatory period of force-matching.

### Enhanced efficacy of information transfer for the increased postural challenge

In light of the global and local efficiencies (*E*_*loc*_ and *E*_*glob*_; Figure 3, the third row), the information transfer in the brain network was significantly enhanced in the stabilometer condition. Analogous to difficult manipulation of a mathematical task (Klados et al., 2013), the information transfer in brain networks for a postural-suprapostural task consistently increased with stance difficulty for all SL thresholds. The stance-related increase in *E*_*loc*_ reflects more short-range connections between neighboring brain regions (particularly in the frontal and premotor areas), by virtue of the high clustering coefficients during concurrent force-matching from stabilometer stance (Figure 5). This increasing nodal organization was compelling evidence of context-dependent recruitment of the local frontal area with high postural demands (Mihara et al., 2008; Huang et al., 2014; Mirelman et al., 2014), which allowed the participants to effectively resolve behavioral interference between the component tasks and to plan the timing of force-matching (Pfurtscheller and Berghold, 1989) under the critical posture condition. On the other hand, an enhanced *E*_*glob*_ indicates a more optimal network architecture for direct information transfer among distributed regions, commonly seen in skill advancement following motor learning (Sami and Miall, 2013). In the stabilometer condition, the long-distance connectivity between the prefrontal/frontal area and the sensorimotor area (Figures 4B, 6B) facilitates the integration of posture-stabilizing information by selectively gating sensory inputs from multiple sources from the sensorimotor area with the central executive function. However, the small-world property (*sigma*) did not vary with stance configuration (Figure 3), indicating a stance-independent balance between local processing specialization and global information propagation.

### Anterior shift in processing resources in the stabilometer condition

The second major finding of this study was that the increase in stance difficulty caused an anterior shift in processing resources for force-matching from stabilometer stance, in support of several lines of evidence, including increasing frontal emphasis of the SL adjacent matrix (Figures 3, 4), potentiation of frontal clustering coefficients (Figure 5B), enhancement of mid-frontal theta connectivity (Figure 6A), and increases in inter-regional coupling from frontal to sensorimotor networks in the upper alpha and beta bands (Figure 6B). These scenarios jointly suggested a transition of the postural-suprapostural task, which is typically regulated by the frontal-parietal executive system in the level-surface condition, to a state in which the frontal strategic control prevails for concurrent force-matching from the stabilometer stance (Slobounov et al., 2009; Ferraye et al., 2014; Karim et al., 2014). The stabilometer stance altered brain resource reallocation in at least three different aspects: recruitment of resources necessary for dealing with the postural instability, target detection for force-matching, and task-switching associated with the increase in postural load. First, the additional recruitment of frontal executive resources was partly attributable to the attentional focus being shifted to postural destabilization due to variations in stabilometer movement (Dault et al., 2001), as previous studies have found from recording cortical activation in the prefrontal cortex, frontal cortex, and supplementary motor area following posture perturbation (Mihara et al., 2008; Fujimoto et al., 2014). Next, stabilometer movement aggravated externally-induced retinal image motion, adding difficulty to the action monitoring and error detection with visual feedback for force-matching (Sipp et al., 2013; Hülsdünker et al., 2015). Therefore, the enhanced mid-frontal theta activity could also reflect heightened selective attention to improve gaze stability for the detection and prediction of target movements (Mihara et al., 2008). Third, for a postural-suprapostural setup with a suprapostural motor goal, the amplitudes of the N1 and P2 components in the preparation period are related to variations in the task-load of the postural and suprapostural tasks, respectively (Huang and Hwang, 2013). This fact clearly suggests that tasks are scheduled in a sequential order to execute a force-matching act with postural prioritization. Since switch costs are greater when switching from a more difficult task to an easier task (Schneider and Anderson, 2010; Barutchu et al., 2013), the switch cost from the postural task to force-matching in the stabilometer condition was multiplied, entailing an extra computational load on frontal executive function (Liefooghe et al., 2008) in the preparatory period. In addition to increases in the SL_F and frontal clustering coefficients, it was of great significance to observe the increases in the connectivity of functionally specialized regions between the left prefrontal area (Fp_1_) and sensorimotor cortex (C_3_, C_4_, C_z_, CP_3_, CP_4_, and CP_z_) in the upper alpha (10–13 Hz) and beta (13–35 Hz) bands (Figure 6B). Such long-distance connectivity well explains the greater global coefficient (*E*_*glob*_) for concurrent force-matching and stabilometer stance (Figure 3), suggesting underlying cooperative activities within the dorsolateral prefrontal cortex, anterior cingulate cortex, and supplementary motor area (Kondo et al., 2004; Hashimoto et al., 2011). Taxing frontal resources for concurrent postural-suprapostural tasks under high postural threats conceptually supports the previous thinking that the prefrontal or frontal network could be a common bottleneck for dual-tasks (Dux et al., 2006). In addition, the overall increase in functional connectivity (SL_all) with a frontal emphasis is direct neurophysiological evidence of increasing stance-related attentional control over a postural-suprapostural task with increasing postural difficulty, hypothetically indexed with entropy measures of the posture component task (Table 1) and previous work (Donker et al., 2007; Stins et al., 2009).

### Brain network economy for postural-suprapostural task with increased postural challenge

The most interesting finding was a strategic trade-off to avoid a resource ceiling by reducing reliance on the temporal-parietal-occipital network, when the stance difficulty increased for a postural-suprapostural dual-task. In a challenging posture such as the stabilometer condition, stronger inter-dependencies among the temporal-parietal junction (Tachibana et al., 2011; Karim et al., 2013) and parietal-occipital areas (Slobounov et al., 2005, 2009; Pellijeff et al., 2006) are expected, as the subjects needed to depend on an enhanced vestibule-ocular response, visual-proprioceptive control, and motion vision to establish dynamic representations of body schema due to postural destabilization. However, in view of the lower SL_PO (Figures 3, 4) and parietal clustering coefficients (Figure 5), the present study conversely exhibited desynchronization of the parietal-occipital network. Moreover, the spectral connectivity of the upper alpha and beta bands in the temporal-parietal-occipital regions (particularly in the right hemisphere) was suppressed during concurrent force-matching from a stabilometer (Figure 6B). These facts suggest neural economy to prevent excessive consumption of brain resources, affected by restricting the division of attentional resources toward multisensory information before the force-matching act from the stabilometer stance. It seems that the visuospatial attention to postural control to detect enhanced postural fluctuations could be temporarily disengaged during preparation to execute a force-matching task. We argue that the information inhibition is potentially advantageous in that it increases the resources allocated to frontal executive function, empowering conflict detection, task switching, and facilitation of the suprapostural goal. Due to the flexible resource allocation, the accuracy and responsiveness of force-matching were not affected by postural destabilization (Table 1). Supporting the notion of a domain-specific idling of dorsal networks to prevent a resource ceiling, previous neuroimaging studies also reported a comparable neural economy under the condition of relatively high postural threats. Imaged locomotion produced lower activity in the vestibular and somatosensory (right hemisphere preponderance) areas than that during imagined standing (Jahn et al., 2004; Zwergal et al., 2012). However, it should be noted that resource allocation for a dual-task is context dependent. During the concurrent execution of two verbal tasks, Mizuno et al. (2012) found no significant connectivity changes in the parietal and temporal areas with increases in task load. Classic dual task setups (cognitive or verbal tasks) might have very different task compatibilities and reciprocal effects from a postural-suprapostural task (Huang and Hwang, 2013), since a postural subtask is always prioritized naturally, consuming a variety of brain resources.

### Methodology issue

The execution of postural-suprapostural tasks must rely on coordination interactions of neuronal sources across distributed brain regions. Among the several quantitative approaches, SL was used to characterize the inter-dependences between two cortical activities because it is the most popular index for estimating functional connectivity for neurophysiological data. SL has been widely been used to assess connectivity strength in the graphic based studies using either low-density (Pijnenburg et al., 2004; Smit et al., 2012; Boersma et al., 2013; Liu et al., 2015; Herrera-Díaz et al., 2016) or high-density EEG (Polanía et al., 2011; Cao et al., 2014), because SL is able to account for the repertoire of network states, considering linear and nonlinear interactions between multiple synchronized neural sources in the brain (Stam and van Dijk, 2002). Also, SL can sensitively detect slight and complex variations in the coupling strength (Koenis et al., 2013) and resolve synchronization patterns on a fine time scale (Stam and van Dijk, 2002; Betzel et al., 2012). These advantages were especially helpful in highlighting stance-related differences in rapid dynamic of the ERP with low-frequency oscillations (such frontal theta synchronization and long-stance upper alpha desynchronization; Figures 6A,B). However, a few researchers based on stimulation studies to argue that SL is not immune to a volume conduction effect that might cause spurious coupling from common diploe sources (Stam et al., 2005; Tognoli and Kelso, 2009). If the physical synchronization did exist during the experiment, we could not conclusively deny overestimation of the observed differences in local clustering schema, since correlations generated from single diploes tend to be strongest within neighboring electrodes. Despite a potential volume conduction effect, physical synchronization did not rationally explain the patterned changes in network connectivity of the frontal, sensorimotor, and parietal-occipital networks (Figures 4B, 5B), as a global rise or fall of state transition was not influenced by intermittent activity of a single common source. In addition, the stance-dependent changes in network properties of spatially distributed communities was observed across the threshold values (Figure 3), which are hard to reconcile with known spatial influences due to volume conduction. To date, there is no perfect mathematical tool to assess inter-regional connectivity. Although some measures of inter-regional connectivity have been proposed to counter common sources like phase lag index (Stam et al., 2007b), yet these phase-based approaches actually measures different inter-dependence properties of regional EEG signals as the SL. In fact, phase-based approaches can be more susceptible to small perturbations (Vinck et al., 2011), raising another validity issue to identify ERP connectivity in the presence of noise and non-stationarities (Cohen, 2015). Therefore, a further study may consider EEG recordings simultaneously with BLOD activation patterns for methodological exactness. However, it is beyond the scope in this study to utterly preclude hypothetical common source with the present setup.

Next, in addition to stimulus-locked ERP used in this study, an alternative analysis is to lock EEG activity with force-matching event (response-locked ERP). The selection of time-lock depends on the experimental design to highlight different information processing underlying response preparation. The response-lock approach is recommended, if the experiment does not change premotor processing (Mordkoff and Gianaros, 2000). When response preparation varies with experimental manipulation, information processing in the time domain is stretched or compressed with the use of response-locked ERP. As reaction time of force-matching was hypothesized to vary with the effect of stance configuration, we favored stimulus-locked ERP to assess dual-task effects in this study. Analysis using response-locked ERP seems to be more popular in those single motor task experiments.

## Conclusions

With brain connectivity analysis, the present work highlights the availability of adaptive resource allocation to explain concurrent suprapostural performance that is insusceptible to increasing postural load. Theoretical graphic analysis validated the hypothesis that brain reorganization would lead to a functional network with superior efficacy and global information transfer to cope with increasing stance difficulty for a postural-suprapostural task. In addition, we have identified fine-grained details regarding cost-effective mechanisms for this particular dual-task condition, involving an anterior shift to frontal processing resources and dorsal idling of the parietal-occipital networks.

## Author contributions

Substantial contributions to the conception or design of the work; or the acquisition, analysis, or interpretation of data for the work: Conception or design of the work, CH, IH; Acquisition, YT; Analysis, GC, IH; Interpretation of data, CH, IH. Drafting the work or revising it critically for important intellectual content: CH, GC, YT, IH. Final approval of the version to be published: CH, GC, YT, IH. Agreement to be accountable for all aspects of the work in ensuring that questions related to the accuracy or integrity of any part of the work are appropriately investigated and resolved: CH, GC, YT, IH.

## Funding

This research was supported by grants from the Ministry of Science and Technology, R.O.C. Taiwan, under grant no. MOST 103-2314-B-002 -007 -MY3 and MOST 104-2314-B-006 -016 -MY3.

### Conflict of interest statement

The authors declare that the research was conducted in the absence of any commercial or financial relationships that could be construed as a potential conflict of interest.

## Abbreviations

SampEn: sample entropy
SL: synchronization likelihood
*C*_*w*_: clustering coefficient
*E*_*glob*_: global coefficient
*E*_*loc*_: local coefficient.
